# The ACURATE *neo*2 valve system for transcatheter aortic valve implantation: 30-day and 1-year outcomes

**DOI:** 10.1007/s00392-021-01882-3

**Published:** 2021-06-20

**Authors:** Helge Möllmann, David M. Holzhey, Michael Hilker, Stefan Toggweiler, Ulrich Schäfer, Hendrik Treede, Michael Joner, Lars Søndergaard, Thomas Christen, Dominic J. Allocco, Won-Keun Kim

**Affiliations:** 1 Department of Internal Medicine I, St.-Johannes-Hospital Dortmund, Johannesstraße 9-13, 44137 Dortmund, Germany; 2 Department of Cardiovascular Surgery, Heart Center Leipzig, Leipzig, Germany; 3 Klinik Für Herz-, Thorax und Herznahe Gefäßchirurgie, Universität Regensburg, Regensburg, Germany; 4 Department of Cardiology, Luzerner Kantonsspital | LUKS, Luzern, Switzerland; 5 Center for Internal Medicine, Marienkrankenhaus, Hamburg, Germany; 6 Department of Cardiovascular Surgery, University Hospital Mainz, Mainz, Germany; 7 Deutsches Herzzentrum München, Technische Universität München, Munich, Germany; 8 DZHK (German Centre for Cardiovascular Research), Munich Heart Alliance, Munich, Germany; 9 Department of Cardiology, Rigshospitalet, Copenhagen, Denmark; 10 Boston Scientific, Marlborough, MA USA; 11 Department of Cardiology/Cardiac Surgery, Kerckhoff Heart Centre, Bad Nauheim, Germany

**Keywords:** Aortic valve stenosis, Transcatheter aortic valve replacement, Transfemoral aortic valve implantation, Paravalvular regurgitation

## Abstract

**Background:**

Transcatheter aortic valve implantation (TAVI) has become standard treatment for elderly patients with symptomatic severe aortic valve stenosis. The ACURATE neo AS study evaluates 30-day and 1-year clinical and hemodynamic outcomes in patients treated with the ACURATE *neo*2 valve.

**Methods:**

The primary endpoint of this single-arm multicenter study is 30-day all-cause mortality. Other key endpoints include device performance, echocardiographic measures assessed by an independent core laboratory, and VARC-2 clinical efficacy and safety endpoints through 12 months.

**Results:**

The study enrolled 120 patients (mean age 82.1 ± 4.0 years; 67.5% female, mean baseline STS score 4.8 ± 3.8%). The VARC-2 composite safety endpoint at 30 days occurred in 13.3% of patients. All-cause mortality was 3.3% at 30 days and 11.9% at 1 year. The 30-day stroke rate was 2.5% (disabling stroke 1.7%); there were no new strokes between 30 days and 12 months. The rate of permanent pacemaker implantation was 15.0% (18/120) at 30 days and 17.8% (21/120) at 1 year. No patients required re-intervention for valve-related dysfunction and there were no cases of valve thrombosis or endocarditis. Patients demonstrated significant improvement in mean aortic valve gradient (baseline 38.9 ± 13.1 mmHg, 1 year 7.8 ± 3.5 mmHg; *P* < 0.001 in a paired analysis). In the overall population, paravalvular leak was evaluated at 1 year as none/trace in 60.5%, mild in 37.0%, and moderate in 2.5%; no patients had severe PVL.

**Conclusions:**

One-year outcomes from the ACURATE neo AS study support the safety and performance of TAVI with the ACURATE *neo*2 valve.

**Graphic Abstract:**

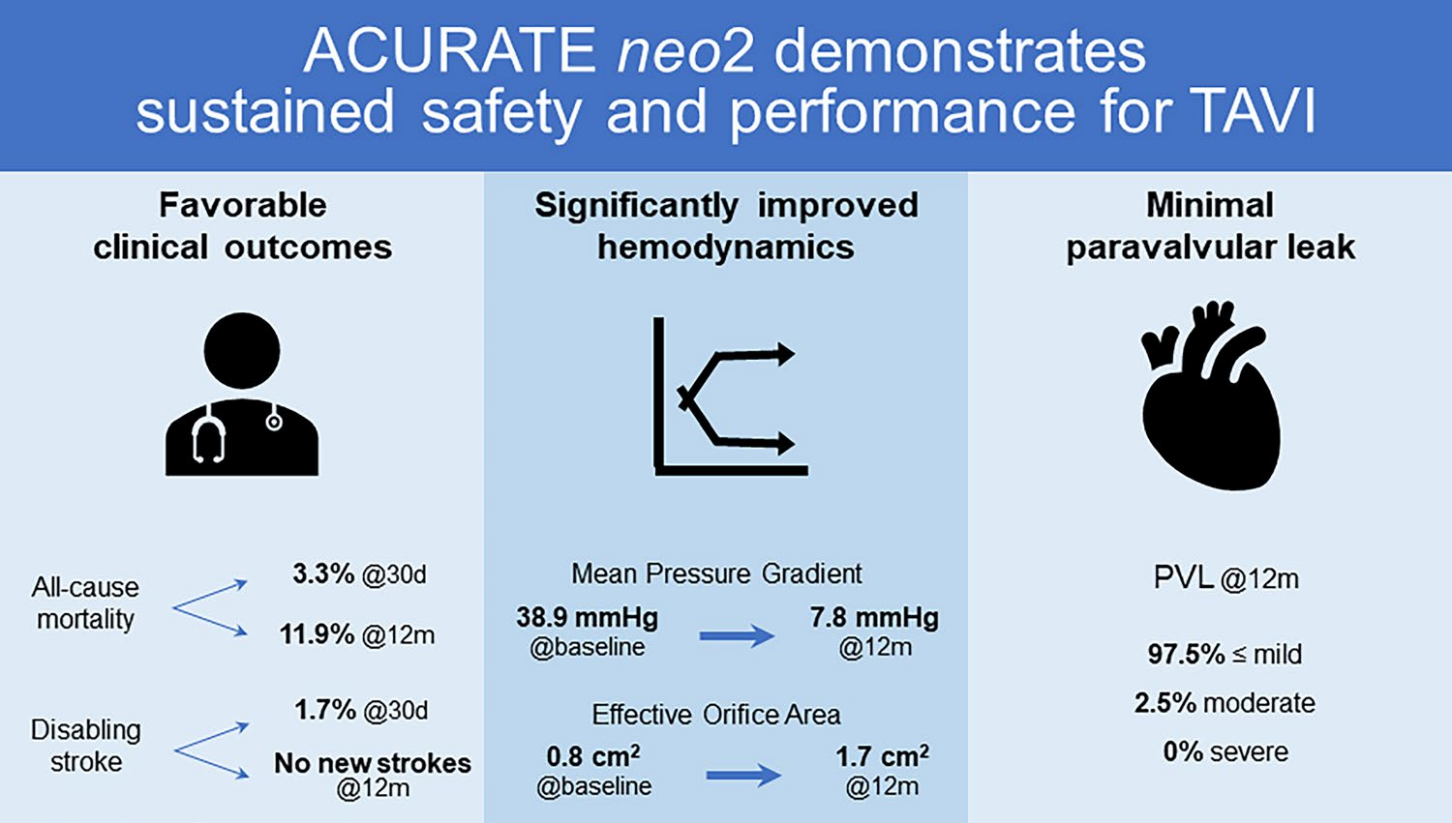

**Supplementary Information:**

The online version contains supplementary material available at 10.1007/s00392-021-01882-3.

## Introduction

Transcatheter aortic valve implantation (TAVI), once reserved for patients who were inoperable or at high risk for surgical valve replacement, has recently been extended to intermediate- or low-risk populations. While clinical outcomes following TAVI are often comparable to those achieved surgically [1–4], there is some evidence that post-TAVI complications such as patient–prosthesis-mismatch (PPM), paravalvular leak (PVL), and permanent pacemaker implantation (PPI) are associated with increased long-term mortality [5–8]. The self-expanding ACURATE *neo* valve (Boston Scientific, Marlborough, MA) was designed to mitigate the risk of some of these complications: the supra-annular leaflet positioning contributes to lower gradients, the lower crown protrudes only minimally into the left ventricular outflow tract (LVOT) to minimize conduction system interference, and the integrated internal and external porcine pericardium sealing skirts reduce PVL [9]. ACURATE *neo* has demonstrated favorable clinical and echocardiographic outcomes, with low rates of mortality and PPI [10, 11]. However, two recent investigator-initiated studies which randomized patients to ACURATE *neo* versus a later-generation competitor device (Sapien 3 and EvolutR/PRO) found a higher incidence of moderate or greater PVL in patients treated with ACURATE *neo*, which contributed to its missing the non-inferiority primary endpoints [12, 13].

**Fig. 1:**
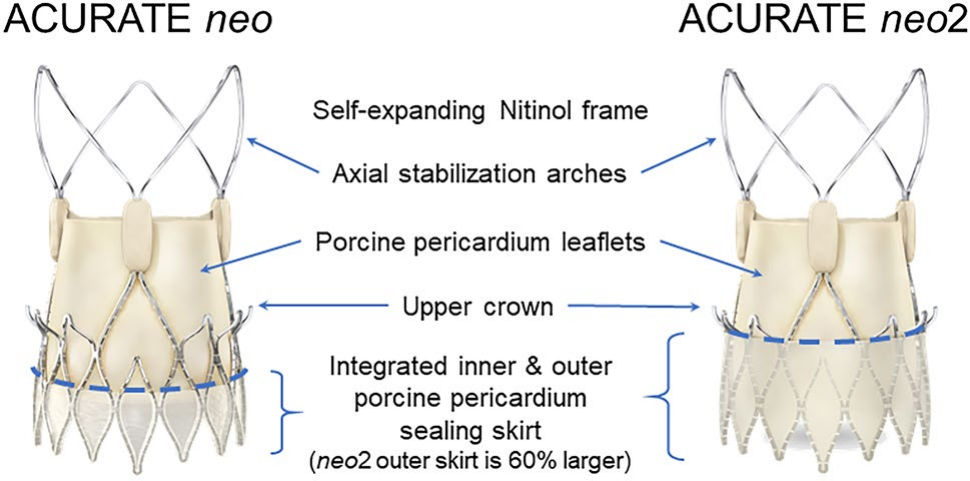
The ACURATE valve family. ACURATE *neo* and ACURATE *neo*2 are transcatheter self-expanding bioprosthetic aortic valves comprised of a nitinol frame with axial, self-aligning stabilization arches and supra-annular porcine pericardium leaflets. ACURATE *neo*2 represents an evolution of the valve design in that it features an enhanced sealing skirt to further reduce paravalvular leak

The ACURATE *neo*2 valve is an evolution of the ACURATE *neo* valve (Fig. 1). The simplified implant procedure and supra-annular valve positioning are preserved, while the sealing skirts have been augmented to further reduce the PVL rate. Here we report the results of the ACURATE neo AS study, which focused on clinical and core laboratory-assessed echocardiographic outcomes after 30 days and 12 months in patients with severe symptomatic aortic stenosis treated with the next-generation ACURATE *neo*2 valve.

## Methods

### Study design and device details

The ACURATE neo AS study was a single arm, prospective, non-randomized study conducted at European centers (see Supplementary Table S1). The study enrolled patients with severe symptomatic aortic stenosis for whom conventional aortic valve replacement was considered high risk for mortality or who were not operable as determined by a heart team consisting of a cardiologist and a surgeon. The study excluded patients with bicuspid aortic valves or previously implanted aortic bioprosthetic valves. Full inclusion and exclusion criteria are detailed in Supplementary Table S2. The protocol was approved by the locally appointed institutional review boards/ethics committees. The study was registered at ClinicalTrials.gov (NCT02909556) and was conducted in accordance with the International Conference for Harmonization Good Clinical Practice (ICH-GCP) regulations and guidelines and the ethical principles outlined in the Declaration of Helsinki. All patients gave written informed consent.

Like its predecessor, the CE-marked ACURATE *neo* aortic bioprosthesis, the ACURATE *neo*2 valve is a transcatheter self-expanding bioprosthetic aortic valve comprised of a nitinol frame with axial, self-aligning stabilization arches and featuring supra-annular porcine pericardium leaflets (Fig. 1). The integrated internal and external porcine pericardium sealing skirts are designed to conform to irregular calcified anatomy with the goal of reducing PVL; the outer skirt on ACURATE *neo*2 has been extended and is 60% larger than the skirt on ACURATE *neo*. The valve prosthesis is available in three different sizes (S: 21 mm ≤ annulus diameter ≤ 23 mm, M: 23 mm < annulus diameter ≤ 25 mm, and L: 25 mm < annulus diameter ≤ 27 mm). In this study, the size L valve was only available after enrollment of the first 30 patients had been completed. Valve sizing was assessed by computerized tomography (CT); final size selection was at the operators’ discretion.

### Clinical endpoints and outcomes analyses

The primary endpoint of the study was the incidence of all-cause mortality in the intent-to-treat (ITT) population at 30 days. Key secondary endpoints included the rate of clinical events as defined per Valve Academic Research Consortium (VARC)-2 guidelines [14] at discharge/7 days, 30 days, and 12 months, and the VARC-2 safety composite at 30 days. Hemodynamic function, including effective orifice area, mean transprosthetic gradient, and aortic regurgitation, were assessed at discharge/7 days, 30 days, and 12 months of follow-up. Device and procedural success were also evaluated (see Supplementary Table S3 for definitions), in addition to functional improvement from baseline as per New York Heart Association (NYHA) Functional Classification.

To evaluate possible structural valve dysfunction, a post hoc evaluation of patients’ longitudinal change in valve hemodynamics between 30 days and 1 year was performed (discharge/7-day data were used if 30-day data were not available). Criteria for hemodynamic valve dysfunction (HVD) were adapted from the recently published VARC-3 standardized definitions [15]. Morphological valve deterioration (Stage 1) is not reported here, as these data were not systematically collected in the ACURATE neo AS study. The definition of moderate HVD (Stage 2) is as follows, with changes for severe HVD (Stage 3) in brackets: increase in mean transvalvular gradient ≥ 10 mmHg {≥ 20 mmHg} resulting in mean gradient ≥ 20 mmHg {≥ 30 mmHg} with concomitant decrease in EOA ≥ 0.3 cm^2^ or ≥ 25% {≥ 0.6 cm^2^ or ≥ 50%}and/or decrease in DVI ≥ 0.1 or ≥ 20% {≥ 0.2 cm^2^ or ≥ 40%}, OR new occurrence or increase of ≥ 1 grade {≥ 2 grades} of transvalvular aortic regurgitation resulting in ≥ moderate {severe} transvalvular aortic regurgitation.

An independent Data Monitoring Committee (DMC) was initially responsible for review of aggregate safety data up to 12 months; in April 2018 an independent Clinical Events Committee (CEC) assumed responsibility for adjudication of all reported VARC-2 endpoint events. All VARC-2 safety events were 100% monitored. To minimize bias and inconsistencies, all available echocardiographic data at baseline, discharge, and 30-day and 12-month follow-up were evaluated by an independent core laboratory (MedStar Health Research Institute, Hyattsville, MD).

### Statistical methods

The study employed an optimal two-stage design, with sample size calculations based on an expected 30-day mortality rate of 10%, based on literature review, and a one-sided alpha of 5%. If three or more deaths occurred in the first stage of the study (*n* = 30 patients), the study could be terminated by the DMC. Per protocol, 30-day safety analyses were performed in the ITT population, which includes all enrolled patients in whom valve implantation was attempted. Clinical outcomes at 12 months were evaluated in those patients who received an ACURATE *neo* valve. Echocardiographic paired analyses were performed in the cohort of patients with core laboratory-adjudicated data available at baseline, 30 days, and 12 months post procedure.

Baseline and outcome variables were summarized using descriptive statistics where appropriate. For the comparison of categorical variables, statistical differences were assessed using a Chi-squared test or a Fisher’s exact test, as appropriate. For the comparison of continuous variables, the Student’s t test or analysis of variance was used. All statistical analyses were two-sided with an alpha level of 5%. Statistical analyses were performed with SAS software (SAS Institute Inc., Cary, NC), version 9.3 or later.

## Results

### Study population

The study enrolled 120 patients between December 2016 and November 2017 at nine European centers. All patients were implanted with the ACURATE *neo*2 Aortic Valve System, so that the ITT and implanted populations were the same (in two patients an ACURATE *neo*2 valve was initially implanted, but the patients subsequently underwent valve-in-valve implantation with a non-study valve). Clinical follow-up data at 30 days were available for 98.3% of enrolled patients (118/120) and 12-month follow-up data were available for 92.5% (111/120). Two patients withdrew consent prior to 30-day follow-up, and an additional seven patients withdrew from the study between 30 days and 12 months.

Study patients were generally representative of patients treated in European contemporary practice (Table 1). The mean age of the study population was 82.1 years and the majority (67.5%) were female. The mean EuroSCORE II was 4.7 ± 3.8% and the mean STS score was 4.8 ± 3.8%; 11.7% of patients had an STS score ≥ 8%. Eight patients (6.7%) had a pacemaker at baseline, and a conduction abnormality was present at baseline in 47.5% of patients. Nearly all patients (119/120; 99.2%) were classified as NYHA Functional Class III or IV at baseline, and 69.2% had a history of coronary artery disease. Mean AV gradient at baseline was 40.3 ± 14.1 mmHg and the mean aortic valve area (effective orifice area, EOA) was 0.74 ± 0.2 cm^2^.

**Table 1:** Patient demographics and baseline characteristics

Variable	*N* = 120
Age, years	82.1 ± 4.0
Gender, female	67.5 (81)
*Risk assessments*	
STS Score, %	4.8 ± 3.8
STS score ≥ 8%	11.7 (14)
EuroSCORE II	4.7 ± 3.8
NYHA Class III or IV	99.2 (119)
*Medical history*	
COPD, moderate or severe	10 (12)
Diabetes mellitus, medically treated	27.5 (33)
History of coronary artery disease	69.2 (83)
Porcelain aorta	5.8 (7)
History of cerebrovascular disease	3.3 (4)
Prior stroke / TIA	10.8 (13)
History of atrial fibrillation	25.0% (30)
*Previous cardiovascular interventions*	
Prior PTCA	4.2 (5)
Prior PTCA with stenting	26.7 (32)
Prior CABG	5.8 (7)
Prior implanted pacemaker	6.7 (8)
*Conduction abnormality at baseline*	
Any conduction abnormality	47.5% (57)
AV block, 1st degree	15.0% (18)
LBBB	10.8% (13)
RBBB	8.3% (10)
*Echocardiographic measurements (core laboratory adjudicated)*	
Aortic valve area (effective orifice area), cm^2^	0.74 ± 0.2
Mean aortic valve gradient, mmHg	40.3 ± 14.1
Peak aortic valve gradient, mmHg	65.9 ± 21.4
Left ventricular ejection fraction, %	55.8 ± 10.1
Aortic regurgitation ≥ moderate^a^	6.1 (7/115)
Mitral regurgitation ≥ moderate^b^	8.3 (9/108)

Values are mean ± standard deviation (*n*) or % (*n*)

*AV* atrioventricular, *CABG* coronary artery bypass graft, *COPD* chronic obstructive pulmonary disease, *LBBB* left bundle branch block, *NYHA* New York Heart Association, *PTCA* Percutaneous transluminal coronary angioplasty, *RBBB* right bundle branch block, *STS* Society of Thoracic Surgeons, *TIA* transient ischemic attack

^a^Evaluated as ‘moderate’ in four patients (3.5%) and ‘moderately severe’ in three patients (2.6%)

^b^Evaluated as ‘moderate’ in seven patients (6.5%) and ‘severe’ in two patients (1.9%)

Per protocol, patients were to be prescribed dual anti-platelet therapy (DAPT) for 6 months post-TAVI, and aspirin for life; anticoagulation therapy was administered according to the usual practice at each site. At discharge, 56% of patients were on DAPT and 44% were taking anticoagulants. DAPT usage was 53% at 30 days and 28.0% at 6 months; anticoagulant usage was 36% at 30 days and 20% at 6 months.

### Clinical outcomes

The median total procedure time was 48.5 min. The most commonly implanted valve size was M (45% of cases). Balloon pre-dilatation was performed in 95.8% of patients; post-dilatation was performed in 32.5%. The rate of procedural success was 97.5% (117/120). Although a single study valve was implanted in every patient, due to an inability to properly seat the valve in the annulus in two cases (one valve embolization and one valve dislodgement/migration) a second non-study transcatheter valve was used (ie, valve-in-valve implantation); data from these patients are included in the 30-day safety analysis, but not the 1-year analyses. In one patient, post-dilatation resulted in ventricular septal perforation and conversion to open heart surgery was necessary. There were no periprocedural deaths. Two patients (1.7%) experienced disabling stroke prior to hospital discharge. There were no instances of coronary obstruction or cardiac tamponade in the periprocedural period, and no patients experienced a periprocedural (≤ 72 h post-procedure) myocardial infarction. Additional procedural details are presented in Table 2.

**Table 2:** Procedural characteristics and periprocedural outcomes

Measure	*N* = 120
Valve size implanted	
S	25.8 (31)
M	45.0 (54)
L^a^	29.2 (35)
Total procedure time, minutes	48.5 [22.5]
Time from femoral insertion to withdrawal of delivery system, minutes	3.0 [1.0]
Balloon pre-dilatation performed	95.8 (115)
Maximum balloon diameter, mm	23.0 [4.0]
Number of balloon inflations	
1	93.0 (107)
2	5.2 (6)
3	1.7 (2)
Post-dilatation performed	32.5 (39)
Maximum balloon diameter, mm	23.0 [2.0]
Number of balloon inflations	
1	84.6 (33)
2	15.4 (6)
Procedural success	97.5 (117)
Valve malpositioning (including valve migration, valve embolization, ectopic valve deployment)^b^	1.7 (2)
Ventricular septal perforation^c^	0.8 (1)
Coronary obstruction	0.0 (0)
Cardiac tamponade	0.0 (0)
MI ≤ 72 h post-procedure	0.0 (0)
Life-threatening or disabling bleeding	5.0 (6)
Disabling stroke	1.7 (2)

Values are % (*n*/120) or median [IQR]

*MI* myocardial infarction

^a^Size L valve was only available in the second phase of enrollment (i.e., after enrollment of the first 30 patients)

^b^Valve-in-valve implantation of a non-study valve required due to valve dislodgement/migration (*n* = 1), valve embolization (*n* = 1)

^c^Perforation resultant from post-dilatation; patient was converted to open heart surgery

The rate of all-cause mortality at 30 days in the ITT population (primary endpoint) was 3.3% (4/120); none of the deaths were valve-related (Table 3). The 1-year all-cause mortality rate was 11.9% (14/118). The stroke rate was 2.5% at 30 days (3/120; periprocedural disabling stroke in two patients, and one additional non-disabling stroke on day 5). There were no additional stroke events between 30 days and 12 months. Major vascular complications occurred in four patients (3.3%) through 30 days. The VARC-2 composite safety endpoint, which includes all-cause mortality, all stroke, major vascular complications, life-threatening or disabling bleeding, acute kidney injury (Stage 2/3), repeat procedure for valve-related dysfunction, and coronary obstruction requiring intervention at 30 days, occurred in 13.3% of patients. No patients in the study required reintervention for valve-related dysfunction, and there were no instances of prosthetic aortic valve thrombosis or endocarditis through 12-month follow-up.

**Table 3:** Safety outcomes

Clinical event	30 Days *N* = 120	1 Year *N* = 118
VARC-2 early safety composite^**a**^	13.3 (16)	–
All-cause mortality	3.3 (4)	11.9 (14)
Cardiovascular mortality	3.3 (4)	9.3 (11)
All stroke	2.5 (3)	2.5 (3)
Disabling stroke	1.7 (2)	1.7 (2)
Major vascular complications	3.3 (4)	3.3 (4)
Life-threatening/disabling bleeding	5.0 (6)	8.5 (10)
Acute kidney injury (stage 2 or 3)	0.8 (1)	0.8 (1)
Myocardial infarction > 72 h	0.8 (1)	0.8 (1)
Repeat procedure (surgery/interventional) for valve-related dysfunction	0.0 (0)	0.0 (0)
Hospitalization for valve-related symptoms or CHF	–	4.2 (5)
New permanent pacemaker	15.0 (18)	17.8 (21)
New-onset atrial fibrillation or atrial flutter	5.8 (7)	8.5 (10)
Coronary obstruction requiring intervention	0.8 (1)	0.8 (1)
Prosthetic aortic valve thrombosis	0.0 (0)	0.0 (0)
Prosthetic aortic valve endocarditis	0.0 (0)	0.0 (0)

Values are % (*n*); two patients required a valve-in-valve procedure with a non-study valve and thus are not included in the 1-year analyses

*BAV* balloon aortic valvuloplasty; *CHF* congestive heart failure

^a^Includes all-cause mortality, all stroke, major vascular complications, life-threatening or disabling bleeding, acute kidney injury (Stage 2/3), repeat procedure for valve-related dysfunction, and coronary obstruction requiring intervention

At 30-day follow-up, left bundle branch block (LBBB) was reported in 24 patients (23.5%). A total of 18/120 patients (15.0%) received a permanent pacemaker within 30 days (18/112 pacemaker-naïve patients; 16.1%). Among these patients, 8/18 (44.4%) had an underlying conduction disorder at baseline: four patients had right bundle branch block (two of these also presented with first degree AV block), three additional patients had first-degree AV block alone, and one patient had incomplete LBBB. Between 30 days and 12 months, three additional pacemakers were implanted, for a 1-year rate of 17.8% (18.8% among pacemaker–naïve patients). A multivariate analysis did not identify any patient or procedural factors related to pacemaker implantation.

**Fig. 2:**
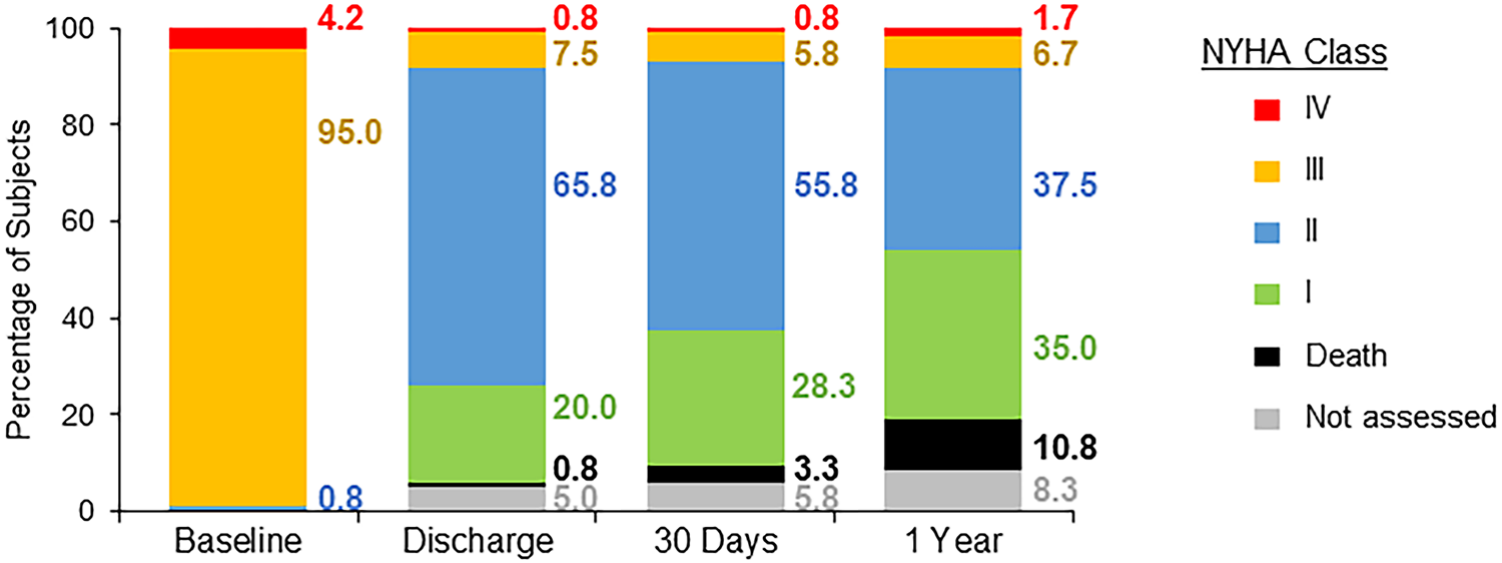
Change in New York Heart Association (NYHA) functional status. Patients exhibited marked improvement in NYHA class at 30 days post-procedure, which was maintained at 1 year

Functional improvement was evaluated per NYHA Functional Classification guidelines. At baseline, 95% of patients were classified as NYHA Functional Class III, and an additional 4.2% were NYHA Class IV (Fig. 2). Patients exhibited substantial improvement in function over the course of the study. From baseline to 12 months post-TAVI, 91% of patients improved at least one functional class and 43% of patients improved at least two classes.

### Echocardiographic outcomes

At 30 days, 115 patients were eligible for transthoracic (TTE) or transeosophageal echocardiography (TEE) assessment (data were unavailable for two patients due to withdrawal from study and three patients due to death prior to follow-up); 30-day echocardiographic data were evaluated for 104/115 patients (90.4%). At 12 months, 98 patients were eligible for echocardiographic follow-up (data unavailable for nine withdrawn patients and 13 deaths); 12-month echocardiographic assessment was completed for 89/98 patients (90.8%).

**Fig. 3:**
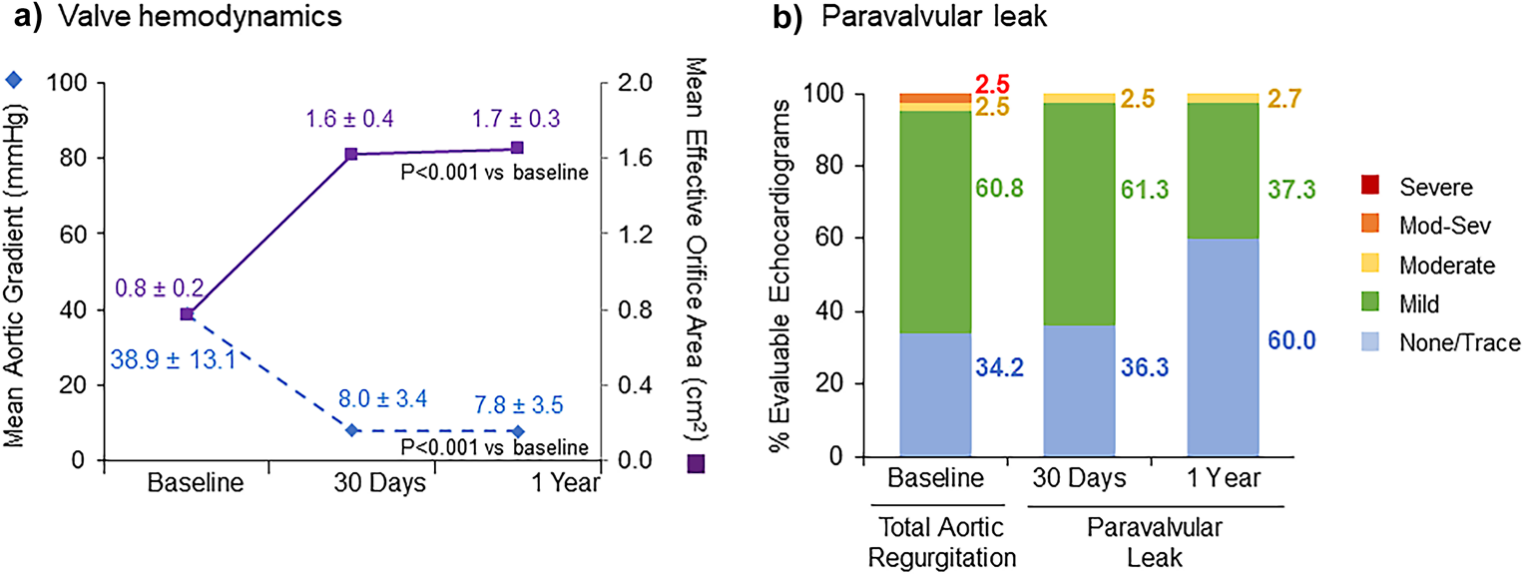
Valve hemodynamics and paravalvular leak. Paired analyses were performed for patients with core laboratory-adjudicated echocardiographic data available at baseline, 30 days, and 12 months (*n* = 80). **a** Both mean aortic valve gradient and mean effective orifice area (reported as time velocity integral [TVI] ratio) significantly improved from baseline to 1 year (*P* < 0.001 for both). **b** Inter-individual improvement in paravalvular leak was observed over the course of the study

The overall as-treated population demonstrated excellent hemodynamics throughout the study (Supplementary Table S4). The mean AV gradient was 7.6 ± 3.5 mmHg and EOA was 1.7 ± 0.4 cm^2^ at 1 year. At 12-month follow-up, 60.5% of the overall study population had no/trace PVL, 37.0% exhibited mild PVL, and 2.5% had moderate PVL. No patients exhibited greater than moderate PVL at any time post procedure. A paired analysis was performed for patients with core laboratory-adjudicated echocardiographic data available at baseline, 30 days, and 12 months (*n* = 80). Patients demonstrated significant inter-individual improvement in mean AV gradient and EOA between baseline and 12 months (*P* < 0.001 for both; Fig. 3a). At 30-day follow-up, PVL was evaluated as none/trace in 36.3% of patients in the paired cohort; this proportion improved to 60.0% at 12 months (Fig. 3b). We evaluated the change in valve hemodynamics between 30 days and 1 year to determine whether any patients had potential structural valve dysfunction. Based on this longitudinal assessment of hemodynamic function, no patients met the criteria for moderate or severe HVD (Supplementary Table S5).

## Discussion

The ACURATE *neo*2 valve preserves many of the desirable attributes of the prior-generation ACURATE *neo* valve, and incorporates new features designed to mitigate some of the common complications associated with TAVI. A flexible delivery catheter allows for trackability through tortuous anatomy, radiopaque markers aid reference in positioning, and a simple two-step, top–down deployment method allows for stable and predictable release. Patients in the ACURATE neo AS study achieved a high rate of procedural success (97.5%). There were no reinterventions for valve-related dysfunction and a low rate of major vascular complications (3.3%), comparable to the rates observed in recent studies with contemporary competitors such as Portico (5.5%) [16], Evolut PRO (3.5%) [17], and Sapien 3 (8.6%) [18]. The ease of use of the ACURATE *neo*2 valve and the operators’ prior experience with the ACURATE platform (over three-quarters of sites/investigators had treated patients with ACURATE *neo* in the SAVI-TF study) may have contributed to the high rate of procedural success observed.

Overall, patients treated with ACURATE *neo*2 exhibited favorable early clinical outcomes. There was a low incidence of disabling stroke at 30 days (1.7%), as in prior studies with ACURATE *neo* (1.2% in SAVI-TF, 1.6% in the NEOPRO study) [11, 17]. The VARC-2 composite safety endpoint rate at 30 days was similar or lower with ACURATE *neo*2 (13.3%) compared with prior studies of ACURATE *neo* (15.8% in the MORENA study [18]; 16.4% in the NEOPRO study [17]; 17.9% in Pellegrini, et al. [19]). All-cause mortality through 1 year was likewise comparable to recent studies with ACURATE *neo* and other contemporary valves [10, 19, 20].

The ACURATE *neo*2 valve maintains a supra-annular leaflet position, allowing for a larger effective orifice area and lower gradients than valves with an intra-annular leaflet position. ACURATE neo AS patients demonstrated marked hemodynamic improvement at 30 days, with a significant change from baseline that was maintained through 12 months of follow-up (Fig. 3). While there have been no studies comparing ACURATE *neo*2 to contemporary competitors, head-to-head comparisons of the prior-generation ACURATE *neo* to Sapien 3 in the MORENA study [18] and by Mauri et al. [20] have shown superior hemodynamics for ACURATE *neo*. Similarly, in the SCOPE I study more favorable gradients and valve areas were recorded for patients treated with supra-annular ACURATE *neo* compared to those treated with the intra-annular SAPIEN 3 device (7 mmHg vs 11 mmHg, *P* < 0.0001; 1.73 cm^2^ vs 1.46cm^2^, *P* < 0.0001) [12].

In spite of its lower gradients and larger valve areas, ACURATE *neo* missed the non-inferiority primary endpoint of early safety and clinical efficacy at 30 days in the SCOPE I study (absolute risk difference 7.1%; upper 95% confidence limit 12.0%; *P* = 0.42), due primarily to a higher incidence of patients with moderate or greater prosthetic valve regurgitation at 30 days (9% vs 3%; *P* < 0.001) [12]. In the SCOPE II study, wherein ACURATE *neo* was randomized against Evolut R/PRO, ACURATE *neo* missed the composite non-inferiority primary endpoint for all-cause death and stroke at 1 year (absolute risk difference 1.8%; upper one-sided 95% confidence limit 6.1%; *P* = 0.05), due to a higher rate of cardiac mortality in the ITT population (8.4% vs 3.9%, *P* = 0.01) [13]. ACURATE *neo* was associated with a higher 30-day rate of moderate or greater PVL (10% vs 3%; *P* = 0.002), which may have contributed to the higher rate of cardiac death. As moderate or greater PVL has been linked to higher mortality rates [21–23], this is a continuing area of concern for TAVI.

The ACURATE *neo*2 valve was designed to improve upon the existing pericardial sealing skirt. The extension of the outer skirt to the waist of the valve enhances the seal, further reducing PVL. Patients in the ACURATE neo AS study exhibited an overall rate of moderate PVL of 3.0% at 30 days, comparable to that observed with the competitor devices in SCOPE I (Sapien 3: 2.8%) and SCOPE II (Evolut: 3.0%). Patients with moderate PVL in the current study had severe calcification at baseline, highlighting the importance of exercising caution in patient selection. In addition to valve design, pre-procedural planning, including determination of optimal sizing and assessment of calcification, is crucial for a good outcome and may help to further lower the incidence of PVL [24]. Longer follow-up is warranted to determine if improvement in PVL translates into improved clinical outcomes.

A low rate of PPI has been a strength of the ACURATE *neo* valve platform. The valve is designed to extend cranially and does not protrude into the LVOT, reducing the risk of conduction system interference. As the overall device specifications and simplified implant procedure are preserved in the ACURATE *neo*2 valve design, it can also be expected to have a low PPI rate. The 30-day PPI rate in the current study (15.0%) is higher than observed with ACURATE *neo* in SAVI-TF (8.3%), or SCOPE I and II (10.0% and 11.0%, respectively). This finding may simply be due to chance, as the sample size analyzed in this manuscript is too small to provide a precise estimate of the PPI risk. Nonetheless, the PPI rate in the current study is within the range observed in recent trials with CoreValve/Evolut (US CoreValve High Risk: 19.8%; Evolut Low Risk: 17.4%; SCOPE II: 18.0%) [1, 13, 25] and Sapien 3 (MORENA: 16.4%; Mauri et al.: 15.2%) [18, 20]. However, such cross-study comparisons should be considered with caution, as the rate of PPI has been shown to vary widely across studies, and a number of factors may contribute to risk [26]. A multivariate analysis of typical risk factors for PPI (including baseline annular calcification, prior conduction disorders, and valve oversizing) did not reveal any strong association. As implanters become more familiar with ACURATE *neo*2 and take steps to optimize implantation technique, refining positioning using radiopaque markers, the PPI rate may decline.

The ACURATE neo AS study has a number of limitations. It was a single-arm, non-randomized study conducted in a relatively small population, and echocardiographic assessment was not available for all patients in the study at all time points, due primarily to differences in follow-up per local standard of care. Additionally, calcification data was qualitative only, and the protocol did not mandate core laboratory assessment of CT data, limiting the ability to assess the impact of annular calcification and valve sizing on clinical outcomes. Perhaps the greatest limitation is the absence of a direct comparator for ACURATE *neo*2. The currently enrolling ACURATE IDE Study (NCT03735667) is a large prospective, multicenter, 1:1 randomized-controlled trial that will provide direct comparative data for ACURATE *neo*2 versus either a balloon-expandable (Sapien 3) or self-expanding (CoreValve / Evolut R / Evolut PRO) prosthetic valve.

## Conclusions

Patients treated with the ACURATE *neo*2 valve demonstrated good early clinical outcomes and showed significant improvement in valve hemodynamics at 30 days, which was maintained through 12-month follow-up. The overall rate of paravalvular leak was low, suggesting an improvement over prior studies with ACURATE *neo*.

## Supplementary Information

Below is the link to the electronic supplementary material.

Supplementary file 1 (DOCX 31 kb)

## Data Availability

The data and study protocol for this clinical trial may be made available to other researchers in accordance with Boston Scientific’s Data Sharing Policy on the Boston Scientific website.
