# Supplementary material for: The ACURATE neo2 valve system for transcatheter aortic valve implantation: 30-day and 1-year outcomes
Source: Clin Res Cardiol. 2021 Jun 20;110(12):1912–20. doi: 10.1007/s00392-021-01882-3 (PMC8639565; doi:10.1007/s00392-021-01882-3)
Supplement: Supplementary file 1 — (DOCX 31 kb) [file 392_2021_1882_MOESM1_ESM.docx]

Supplementary Material Table of Contents

[Table S1. Study Centres and Site Enrolment 2](#_Toc69207513)

[Table S2. ACURATE *neo* AS Study Inclusion and Exclusion Criteria 3](#_Toc69207514)

[Table S3. Secondary Endpoints and Additional Measures 5](#_Toc69207515)

[Table S4. Echocardiographic Outcomes (As-treated population) 6](#_Toc69207516)

[Table S5. Hemodynamic Valve Dysfunction (HVD) 7](#_Toc69207517)

### Table S1. Study Centres and Site Enrolment

| **Institute** | **Site Principal Investigator** | **City, Country** | **Subjects Enrolled** |
| --- | --- | --- | --- |
| Kerckhoff Heart Center, Department of Cardiology | Won-Keun KIM | Bad Nauheim, DE | 35 |
| St.-Johannes-Hospital Klinik für Innere Medizin I | Helge MÖLLMANN | Dortmund, DE | 33 |
| Herzzentrum Leipzig GmbH | David HOLZEY | Leipzig, DE | 20 |
| Universitatsklinik Regensburg, Klinik und Poliklinik fur Herz-, Thorax- und herznahe Gefaßchirurgie | Michael HILKER | Regensburg, DE | 16 |
| Universitäres Herzzentrum Hamburg, Klinik und Poliklinik für Herz- und Gefäßchirurgie | Ulrich SCHAEFER^a^ | Hamburg, DE | 4 |
| Luzerner Kantonsspital | Stefan TOGGWEILER | Luzern, CH | 4 |
| Universitätsklinikum Halle Universitätsklinik und Poliklinik für Herzchirurgie | Hendrik TREEDE^b^ | Halle, DE | 3 |
| Deutsches Herzzentrum München des Freistaates Bayern Klinik für Herz- und Kreislauferkrankungen | Michael JONER | Münich, DE | 3 |
| Rigshospitalet, Kardiologisk klinik | Lars SOENDERGAARD | Copenhagen, DK | 2 |
| **Total** |  |  | **120** |
| ^a^Investigator left site on 30 Spetember 2018 and is currently affiliated with Marienkrankenhaus, Center for Internal Medicine, Hamburg, Germany  ^b^Investigator left site on 30 November 2018 and is currently affliated with Universitätsklinikum Mainz, Mainz, Germany | | | |

### Table S2. ACURATE *neo* AS Study Inclusion and Exclusion Criteria

| **Inclusion Criteria**  *Subjects must have fulfilled all following inclusion criteria in order to be eligible for the study:* |
| --- |
| Aged 75 years and older |
| Severe aortic stenosis defined as: Mean aortic gradient > 40 mmHg *or* Peak jet velocity > 4.0 m/s *or* Aortic valve area of < 1.0 cm2 |
| High risk candidate for conventional AVR defined as: Logistic EuroSCORE 1 ≥ 20% *or* STS Score ≥ 8% *or* Heart team (cardiologist and cardiac surgeon) consensus that patient is not a surgical candidate for conventional AVR due to significant co-morbid conditions unrelated to aortic stenosis |
| NYHA Functional Class > II |
| Aortic annulus diameter from ≥ 21mm up to ≤ 27mm by CT |
| Patient willing to participate in the study and provides signed informed consent |
| **Exclusion Criteria**  *Subjects will be excluded from the study if presenting any of the following:* |
| Congenital aortic stenosis or unicuspid or bicuspid aortic valve |
| Non-stenotic Aortic Insufficiency |
| Severe eccentricity of calcification |
| Severe mitral regurgitation (>2+) |
| Presence of mitral bioprosthesis |
| Presence of previously implanted aortic bioprosthesis |
| Presence of prosthetic ring |
| Anatomy NOT appropriate for transfemoral implant due to the size, disease and degree of calcification or tortuosity of the aorta or ilio-femoral arteries |
| Thoracic (TAA) or abdominal (AAA) aortic aneurysm |
| Presence of endovascular stent graft for treatment of TAA or AAA |
| Trans-oesophageal echocardiogram (TEE) is contraindicated |
| Evidence of intra-cardiac mass, thrombus or vegetation |
| Severe ventricular dysfunction with ejection fraction < 20% |
| Any percutaneous intervention, except for balloon valvuloplasty within 1 month prior to implant procedure |
| Acute Myocardial Infarction within 1 month prior to implant procedure |
| Previous TIA or stroke within 3 months prior to implant procedure |
| Active ulcer or gastrointestinal bleeding within 3 months prior to implant procedure |
| Any scheduled surgical or percutaneous procedure to be performed prior to 30 day visit |
| Severe coagulation conditions |
| Refusal of blood transfusions |
| Systolic pressure <80mmHg, cardiogenic shock, need for inotropic support or IABP |
| Hypertrophic cardiomyopathy with or without obstruction |
| Active bacterial endocarditis or other active infections |
| Hepatic failure (> Child B) |
| Chronic renal dysfunction with serum creatinine > 2.5 mg/dL or renal dialysis |
| Refusal of surgery |
| Severe COPD requiring home oxygen |
| Neurological disease severely affecting ambulation or daily functioning, or dementia |
| Life expectancy < 12 months due to non-cardiac co-morbid conditions |
| Inability to tolerate anticoagulation therapy |
| Contraindication to contrast media or allergy to nickel |
| Currently participating in an investigational drug or another device study |
| Non-valvular aortic stenosis |
| Non-calcific acquired aortic stenosis |
| AVR, aortic valve replacement; COPD, chronic obstructive pulmonary disease; CT, computerized tomography; IABP, intra-aortic balloon pump; NYHA, New York Heart Association; STS, Society of Thoracic Surgeons; TIA, transient ischemic attack |

### Table S3. Secondary Endpoints and Additional Measures

| **Secondary Endpoints** |
| --- |
| Rate of clinical events as defined per VARC guidelines (18) at 7 days / discharge, 30 days and 12 months post procedure:   - Mortality - Stroke - Myocardial infarction - Bleeding complication - Acute kidney injury - Vascular complication - Conduction disturbances and arrhythmia - Other TAVI-related complications |
| Procedural success defined as absence of complications arising during implantation of the prosthetic valve such as: inability to properly seat the valve in the annulus; need for more than one aortic bioprosthesis (valve in valve); or if a surgical aortic valve replacement is required to correct a severe aortic regurgitation or procedure complication. |
| Device success defined as:   - Absence of intra-procedure mortality AND, - Correct positioning of a single prosthetic heart valve into the proper anatomical - location AND, - Intended performance of the prosthetic heart valve (EOAi >0.85 cm2/m2 and mean - aortic valve gradient <20mmHg without moderate or severe AR) |
| VARC composite Safety at 30 days |
| Functional improvement from baseline as per NYHA Functional Classification at 7 days / discharge (whichever occurs first), 30 days and 12 months follow-up |
| Improvement from baseline of the hemodynamic function: effective orifice area, mean transprosthetic gradient at 7 days / discharge (whichever occurs first), 30 days and 12 months follow-up |
| Total aortic regurgitation at 7 days / discharge (whichever occurs first), 30 days and 12 months follow-up |

### Table S4. Echocardiographic Outcomes (As-treated population)

| **Echocardiographic Measure** | **Baseline** | **30 Days** | **1 Year** |
| --- | --- | --- | --- |
| Mean AV gradient, mmHg | 40.3 14.1 (116) | 7.9 3.2 (104) | 7.6 3.5 (85) |
| Mean AV area (effective orifice area), cm^2^ | 0.7 0.2 (108) | 1.7 0.4 (99) | 1.7 0.4 (77) |
| Left ventricular ejection fraction, % | 55.8 10.1 (98) | 57.0 8.4 (81) | 58.2 8.2 (54) |
| Aortic Regurgitation | Total | Paravalvular | |
| None/Trace | 36.0% (41/114) | 35.0% (35/100) | 60.5% (49/81) |
| Mild | 57.9% (66/114) | 62.0% (62/100) | 37.0% (30/81) |
| Moderate | 3.5% (4/114) | 3.0% (3/100) | 2.5% (2/81) |
| Moderate-severe | 2.6% (3/114) | 0.0%  (0/81) | 0.0% (0/81) |
| Severe | 0.0% (0/114) | 0.0% (0/100) | 0.0% (0/81) |

Values are mean ± standard deviation (n) or % (n/N).

### Table S5. Hemodynamic Valve Dysfunction (HVD)

| **ACURATE neo AS HVD at 1 year** | **N=120** |
| --- | --- |
| Moderate HVD (Stage2) | 0.0% (0/69) |
| Increase in mean transvalvular gradient ≥10 mmHg resulting in mean gradient ≥20 mmHg with concomitant decrease in EOA ≥0.3 cm^2^ or ≥25% *and/or* decrease in Doppler velocity index ≥0.1 or ≥20% compared to echocardiographic assessment performed 30-day post-procedure*  *OR* | 0.0% (0/76) |
| New occurrence or increase of ≥1 grade of transvalvular AR resulting in ≥moderate transvalvular AR | 0.0% (0/81) |
| Severe HVD (Stage3) | 0.0% (0/69) |
| Increase in mean transvalvular gradient ≥20 mmHg resulting in mean gradient ≥30 mmHg with concomitant decrease in AVA ≥0.6 cm^2^ or ≥50% and/or decrease in Doppler velocity index ≥0.2 or ≥40% compared to echocardiographic assessment performed 30-day post-procedure*  OR | 0.0% (0/76) |
| New occurrence or increase of ≥2 grades of transvalvular AR resulting in Severe transvalvular AR | 0.0% (0/81) |
| *7-day/discharge data used if 30-day data were not available  Criteria for HVD were adapted from the recently published VARC-3 standardized definitions (Généreux, P, et al. *J Am Coll Cardiol* 2021 Jun 1;77(21):2717-2746). | |
